# Quantification of individual remyelination during short-term disease course by synthetic magnetic resonance imaging

**DOI:** 10.1093/braincomms/fcac172

**Published:** 2022-06-28

**Authors:** Ruth Schneider, Britta Matusche, Theodoros Ladopoulos, Ilya Ayzenberg, Anne Sophie Biesalski, Ralf Gold, Barbara Bellenberg, Carsten Lukas

**Affiliations:** Department of Neurology, St Josef Hospital, Ruhr-University Bochum, 44791 Bochum, Germany; Institute of Neuroradiology, St Josef Hospital, Ruhr-University Bochum, 44791 Bochum, Germany; Department of Neurology, St Josef Hospital, Ruhr-University Bochum, 44791 Bochum, Germany; Department of Neurology, St Josef Hospital, Ruhr-University Bochum, 44791 Bochum, Germany; Department of Neurology, St Josef Hospital, Ruhr-University Bochum, 44791 Bochum, Germany; Department of Neurology, St Josef Hospital, Ruhr-University Bochum, 44791 Bochum, Germany; Institute of Neuroradiology, St Josef Hospital, Ruhr-University Bochum, 44791 Bochum, Germany; Institute of Neuroradiology, St Josef Hospital, Ruhr-University Bochum, 44791 Bochum, Germany; Department of Diagnostic and Interventional Radiology and Nuclear Medicine, St Josef Hospital, Ruhr-University Bochum, 44791 Bochum, Germany

**Keywords:** synthetic MRI, remyelination, diffusion MRI, MTHFR

## Abstract

MRI is an important diagnostic tool for evaluation of myelin content in multiple sclerosis and other CNS diseases, being especially relevant for studies investigating remyelinating pharmacotherapies. In this study, we evaluated a new synthetic MRI–based myelin estimation in methylenetetrahydrofolate reductase deficiency as a treatable primary demyelinating disorder and compared this method with established diffusion tensor imaging in both methylenetetrahydrofolate reductase deficiency patients and healthy controls. This is the first synthetic MRI–based *in vivo* evaluation of treatment-associated remyelination. 1.5 T synthetic MRI and 3 T diffusion MRI were obtained from three methylenetetrahydrofolate reductase deficiency patients at baseline and 6 months after therapy initiation, as well as from age-matched healthy controls (diffusion tensor imaging: *n* = 14, synthetic MRI: *n* = 9). Global and regional synthetic MRI parameters (myelin volume fraction, proton density, and relaxation rates) were compared with diffusion metrics (fractional anisotropy, mean/radial/axial diffusivity) and related to healthy controls by calculating *z*-scores and *z*-deviation maps. Whole-brain myelin (% of intracranial volume) of the index patient was reduced to 6 versus 10% in healthy controls, which recovered to a nonetheless subnormal level of 6.6% following initiation of high-dosage betaine. Radial diffusivity was higher at baseline compared with healthy controls (1.34 versus 0.79 × 10^−3^ mm^2^/s), recovering at follow-up (1.19 × 10^−3^ mm^2^/s). The index patient’s lesion volume diminished by 58% under treatment. Regional analysis within lesion area and atlas-based regions revealed lower mean myelin volume fraction (12.7_Baseline_/14.71_Follow-up_%) and relaxation rates, higher proton density, as well as lower fractional anisotropy and higher radial diffusivity (1.08 × 10^−3^_Baseline_/0.94 × 10^−3^_Follow-up_) compared with healthy controls. The highest *z*-scores were observed for myelin volume fraction in the posterior thalamic radiation, with greater deviation from controls at baseline and reduced deviation at follow-up. *Z*-deviations of diffusion metrics were less pronounced for radial and mean diffusivity than for myelin volume fraction. *Z*-maps for myelin volume fraction of the index patient demonstrated high deviation within and beyond lesion areas, among others in the precentral and postcentral gyrus, as well as in the cerebellum, and partial remission of these alterations at follow-up, while radial diffusivity demonstrated more widespread deviations in supra- and infratentorial regions. Concordant changes of myelin volume fraction and radial diffusivity after treatment initiation, accompanied by dramatic clinical and paraclinical improvement, indicate the consistency of the methods, while myelin volume fraction seems to characterize remyelinated regions more specifically. Synthetic MRI–based myelin volume fraction provides myelin estimation consistent with changes of diffusion metrics to monitor short-term myelin changes on individual patient level.

## Teaser

Schneider *et al*. examined remyelination in methylenetetrahydrofolate reductase deficiency by use of longitudinal myelin estimation based on synthetic MRI, which they compared with metrics derived from diffusion MRI. They suggested that synthetic MRI is suitable to measure short-term myelin changes in CNS diseases on individual patient level.

## Graphical Abstract

**Graphical abstract:**
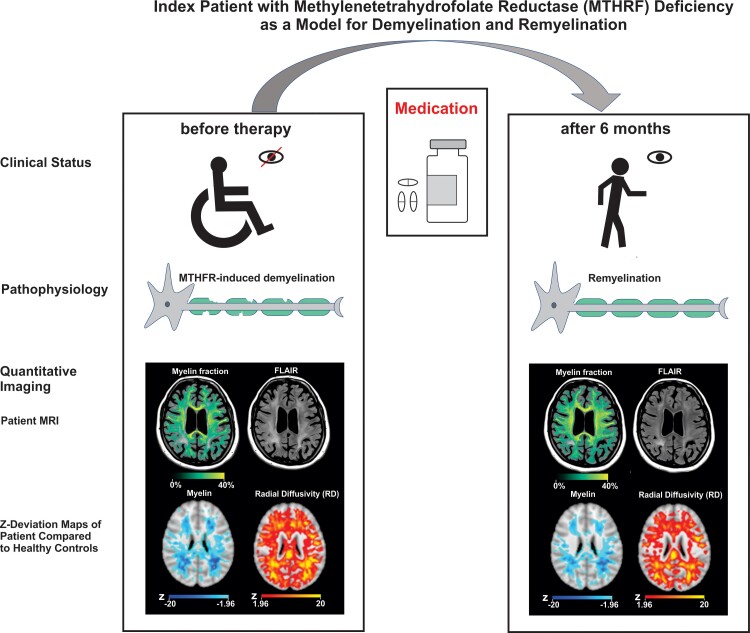

## Introduction

Synthetic MRI (SyMRI) is a novel quantitative imaging technique that is based on a rapid, simultaneous quantification of relaxation times, and proton density (PD), which enables the generation of multiple contrast-weighted images from a single MRI quantification scan.^1^ This 6 min long, multislice, spin-echo sequence allows for automatic, simultaneous brain segmentation and myelin volume measurements.^2^ Recent studies focused on myelin estimation in multiple sclerosis (MS),^3,4^ concluding that SyMRI is a suitable quantitative technique, especially for detecting diffuse demyelination in normal appearing tissue in MS, which is associated with cognitive and clinical disability.^5^ Furthermore, when investigating the characterization of MS plaques, myelin volume fraction (MVF) based on SyMRI results differentiated between plaque or periplaque white matter (WM) and normal appearing WM (NAWM) better than other myelin estimation techniques.^4^

These cross-sectional studies do not allow qualitative conclusions to be drawn about myelin evolution in short-term course at individual patient levels, especially regarding remyelination, which has not been investigated using SyMRI to date (to the best of our knowledge). Therapeutic remyelination is of high research interest for preventing or reversing disease progression in MS.^6,7^ Thus, there is a need for convenient methods of myelin estimations in clinical routine.

In this neuroimaging study, we used a rare autosomal recessive disorder of methionine metabolism as a disease model for visualizing myelin evaluation in short-term course at an individual patient level. The disorder leads to cerebral demyelination but responds with remyelination under treatment.

Methylenetetrahydrofolate reductase (MTHFR) deficiency causes hyperhomocysteinaemia and decreases blood methionine levels. The relationship between decreased S-adenosylmethionine in 5,10-MTHFR deficiency and demyelination in humans was first described by Hyland *et al*. in 1988.^8^ Surtees *et al*.^9^ demonstrated remyelination, showing that following oral l-methionine treatment, there were significant clinical improvements and a complete reversal of the changes in conventional MRI with restoration of normal myelination, which was confirmed by other MRI investigations.^10–12^

We studied an index patient and two of her cousins with MTHFR deficiency to evaluate individual brain myelin changes compared with healthy controls (HCs) and to assess longitudinal changes in myelin volume as a result of treatment medication. A detailed description of the patients’ clinical course has recently been published in a case report by Biesalski *et al*.^13^ Thus, the aims of this study were 2-fold: firstly, to investigate the congruency or complementarity of the novel SyMRI technique in comparison with established diffusion-weighted imaging parameters, and secondly, to identify brain changes at a global, regional, and voxel-based level during the treatment of MTHFR deficiency with regard to de- and remyelination.

## Methods

### Patients and healthy controls

**Figure 1:**
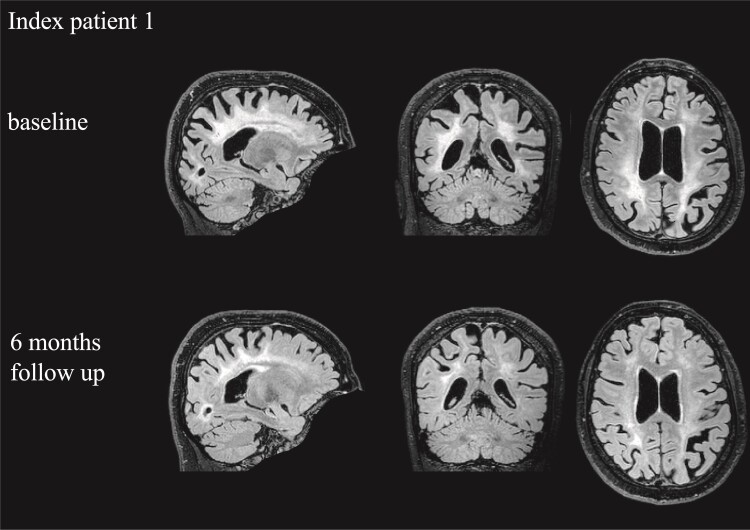
**Temporal change of leucoencephalopathic lesion of the index patient.** Raw 3D T_2_-FLAIR images of the index patient at baseline [upper row; voxel coordinates in each dimension: *x* (left-right) 106, *y* (posterior-anterior) 78, *z* (inferior-superior) 148] and at 6 months follow-up [lower row; voxel coordinates in each dimension: *x* (left-right) 106, *y* (posterior-anterior) 84, *z* (inferior-superior) 164].

Index patient (Patient 1): We investigated brain changes of a 30-year-old female patient suffering from MTHFR deficiency. At the age of 29, the index patient experienced a rapidly progressive and complete loss of vision over 10 weeks and developed severe spastic tetraparesis, ataxia, cognitive impairment, and treatment-refractory seizures. T_2_-FLAIR-weighted MRI (fluid attenuated inversion recovery) revealed symmetrical paraventricular leucoencephalopathy (Fig. 1). A high-dose, oral therapy with betaine, methionine, vitamin B_12_, B_6_, and folic acid was initiated 16 months after manifestation, which resulted in dramatic health improvements over 12 weeks with a complete recovery of vision, and an improvement of cognitive function and gait performance. After 6 months, the patient’s visual acuity remained improved and unassisted walking was possible. In addition, a substantial reduction of the leucoencephalopathic lesion was observed on MRI (Fig. 1).

Further patients with MTHFR deficiency (Patients 2 and 3): The two cousins of the index patient both suffered for considerably longer disease durations than the index patient. Their symptoms included cognitive deficits, movement restrictions forcing them to use crutches or wheelchair, and reduced visual acuity. The initiation of a high-dose betaine therapy, after several years with symptoms, initiated a slight effect on the patients’ visual acuity, while paraparesis and cognitive impairment remained unchanged. We observed leucoencephalopathic lesions on T_2_-FLAIR-weighted MRI for both patients, which were similarly localized, but smaller compared with the index patient (Supplementary Fig. 1). The index patient underwent 1.5 T MRI (SyMRI) and 3 T MRI (diffusion tensor imaging, DTI) before initiation of therapy (baseline) and 6 months later (follow-up), while Patients 2 and 3 only received SyMRI at both time points.

For qualitative and quantitative comparisons, we also studied groups of age-matched, HCs at one time point for DTI (*n* = 14, mean age 28 years) and SyMRI (*n* = 9, mean age 31 years) each. Following the epidemiological characteristics of the index patient, these individuals were selected from pre-existing in-house MRI databases and had received MRI using the same imaging protocols and same scanners for SyMRI (1.5 T) and diffusion-weighted imaging (3 T) as the patients. We included two different groups of HCs, each for the 1.5 T SyMRI and for the 3 T DTI investigations, because the HCs did not receive MRI in both scanners. All included subjects for qualitative and quantitative comparisons were between 25 and 35 years old. The entire pre-existing in-house cohort for the 3 T DTI scans comprised 52 HCs at an age range between 18 and 69 years. The 1.5 T SyMRI cohort involved 39 HCs between the ages of 20 and 68 years.

The Ethical Committee of the Faculty for Medicine at the Ruhr-University Bochum approved the study protocol and written informed consent was obtained from all participants according to the declaration of Helsinki (Reference no. 20-7054-BR).

### Magnetic resonance imaging of the brain

SyMRI imaging was performed using a 1.5 T scanner (Magnetom Aera 1.5; Siemens Healthineers, Erlangen, Germany) with a 16-channel head/neck matrix coil and a multidynamic multiecho sequence, described in previous work^14^ (repetition time: 6930 ms, echo time 1: 23 ms, echo time 2: 102 ms, inversion time: 29 ms, acquisition matrix: 256 × 146, voxel size: 0.9 × 0.9 × 4 mm^3^). The associated SyMRI postprocessing software (Version 11.1.5 for Windows; Synthetic MR, Linköping, Sweden) automatically calculates different parameters and synthetic images: image maps of MVF, R1, and R2 relaxation rates, and of PD. Synthetic T1-weighted images, other image contrasts and an intracranial mask are generated. Furthermore, the software reports global brain measures of total brain volume, GM and WM volumes, intracranial volume (ICV), and myelin volume.

For diffusion-weighted imaging, a 3 T scanner (Achieva; Philips, Best, The Netherlands) with a 32-channel phased-array head coil and a single-shot 2D echo planar imaging sequence with 32 diffusion directions was used (b-factors 0 and 900 s/mm^2^, repetition time: 7 s, echo time: 90 ms, acquisition matrix: 240 × 240, voxel size: 2.5 × 2.5 × 2.5 mm^3^, 50 axial slices). For registration purposes, 3D T1-weighted images were included (repetition time: 10 ms, echo time: 4.6 ms, acquisition matrix: 240 × 240, voxel size: 1 × 1 × 1 mm^3^, 180 axial slices).

### Diffusion MRI preprocessing

Diffusion MRI images of patient and HCs were preprocessed with denoising, Gibb’s ringing correction, bias field correction using MRtrix,^15^ and nonlinear registration of the preprocessed b0 images to high-resolution 3D T1-weighted images to account for susceptibility induced distortions.^16^ Detailed description of the methods can be found in the Supplementary material (Supplementary Figs 2 and 3). The preprocessing steps were performed on both the baseline and the follow-up images, whereby a preceding rigid-body registration step (Functional Magnetic Resonance Imaging of the Brain Software Library (FMRIB, abbreviated FSL) using the linear registration tool, FLIRT)^17,18^ was done for patients’ follow-up scan to align it to the baseline scan.

Subsequently, scalar DTI maps of fractional anisotropy (FA), mean diffusivity (MD), radial diffusivity (RD), and axial diffusivity (AD) were obtained by applying DTIFIT from the FMRIB (Functional MRI of the brain) software library (FSL).^19,20^

The resulting DTI maps were further edited using the first steps of the standard tractography-based spatial statistics (TBSS)^21^ pipeline in FSL.^19,20^ To generate a custom average map as a template representative for HC, all maps of the HC group were averaged using fslmaths to obtain the final group representative parameter maps.

### Synthetic MRI preprocessing

SyMRI maps for both time points (myelin, PD, R1, R2 maps), synthetic T1-weighted images, intracranial masks, and global measures [WM, grey matter (GM), CSF, NON, MVF expressed as % fractions of ICV] were generated automatically using the Synthetic MR Software.^2^ To align the patients’ follow-up images to the baseline maps, FMRIB’s linear and nonlinear registration tools (FLIRT and FNIRT)^19,20^ were used. SyMRI images of the HC group were aligned to the MNI152 template (2 mm resolution)^22^ to create study-specific HC template maps. The preprocessing procedures for SyMRI are extensively described in the supplement.

### Region of interest–based analysis of SyMRI and diffusion metrics

**Figure 2:**
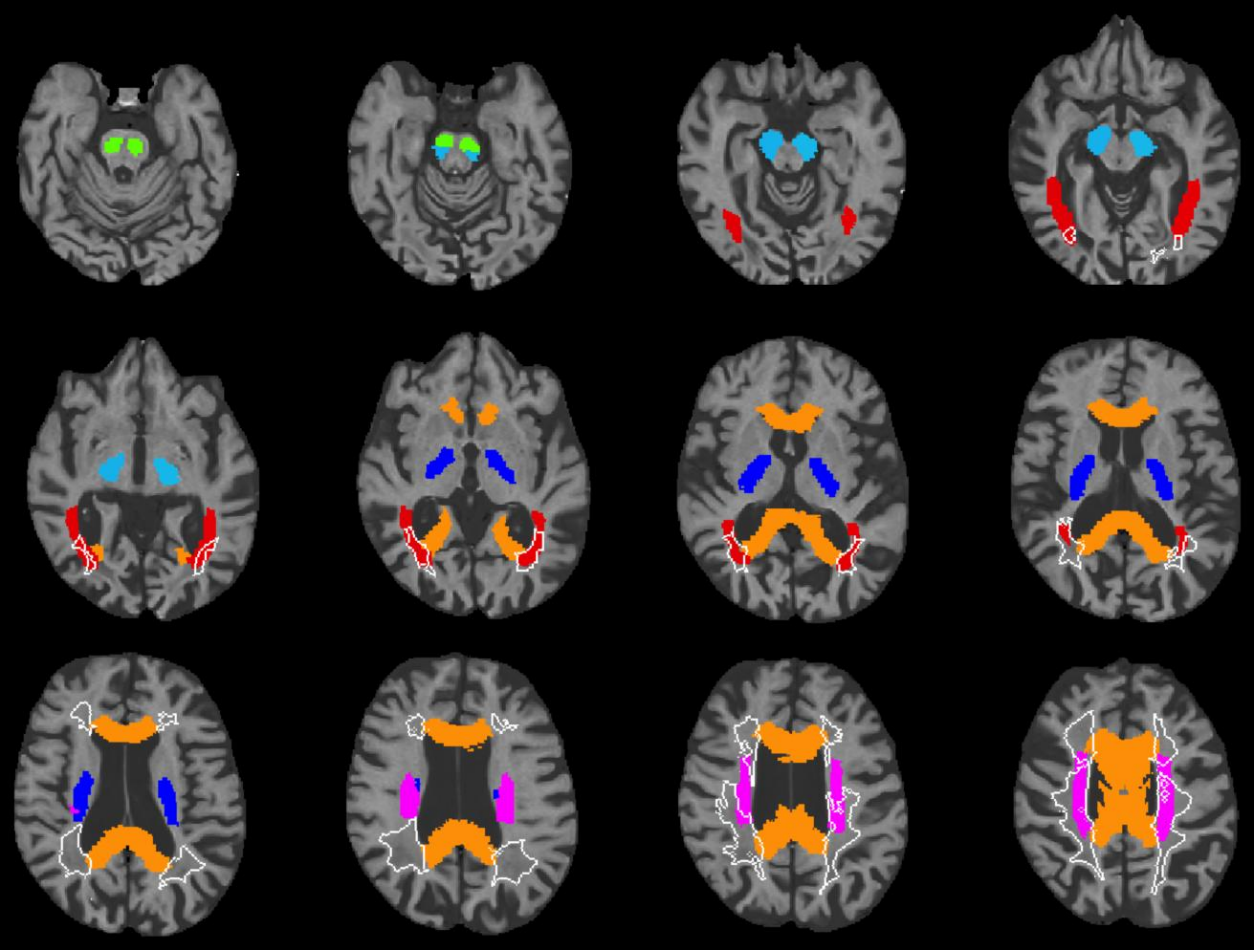
**Regions of interest.** Selected regions of interest with registered lesion mask of Patient 1 at baseline depicted in white. Regions are based on the JHU-ICBM-labels atlas (green = corticospinal tract, lightblue = cerebral peduncle, blue = posterior limb of internal capsule, red = posterior thalamic radiation, orange = corpus callosum, pink = superior corona radiata). Depicted range of *z*-axis (inferior-superior axis): 38–96.

For quantification of DTI metrics, we extracted means and standard deviations of FA, MD, RD, and AD values for the whole brain and within selected regions of interest (ROI) which were derived from the JHU-ICBM-labels atlas^23^ (Fig. 2). Similarly, we extracted ROI-based MVF, R1, R2, and PD from the same atlas ROI. We included regions within major fibre tracts based on leading symptoms of our index patient, which included loss of vision, spastic tetraparesis, and cognitive impairment. Thus, we selected ROI representing the posterior visual tracts and descending motor tracts (the posterior thalamic radiation including optic radiation, corticospinal tract, cerebral peduncle, posterior limb of internal capsule, and superior corona radiata), and the corpus callosum which is known to be involved in cognition by its central function for interhemispheric processing. The corpus callosum was scarcely included in the WM lesion areas in our patients (Fig. 2). For ROI-based parameter extraction, either synthetic T1-weighted or diffusion images were registered to a structural MNI152 template or FMRIB58_FA template using FLIRT and FNIRT, and the resulting warping fields were inverted and applied to the ROI maps. Therefore, mean parameter values of patients and HCs were extracted in individual subject space.

In addition, we extracted quantitative parameters within the patients’ lesion areas affected by the symmetrical paraventricular leucoencephalopathy (Fig. 1). For that, we used SyMRI NON-map of tissue classes that are not classified as WM, GM, or CSF, which is generated automatically by the SyMRI Software, as the basis for lesion maps. We edited the lesion maps manually in accordance with the T_2_ hyperintenties depicted on FLAIR MRI. To compare parameters within this affected area to normal references, the lesion mask was registered to MNI space by applying the warping field from previous registration to the MNI target. Thus, the parameters within the patients’ lesions were compared with the same region in HC by using the patients’ lesion maps for calculation of corresponding HC values. We extracted follow-up parameters using the same baseline lesion mask to investigate longitudinal tissue changes in the region that was affected by leucoencephalopathy at baseline.

### Statistical analysis

The mean values, standard deviations, and confidence intervals (CI = mean ± 1.96 SD) for DTI (FA, MD, RD, AD) and SyMRI metrics (MVF, PD, R1, R2) were obtained from the control group. The individual corresponding metrics of the patients were standardized using *z*-scores:

$$z\text{-}\mathrm{deviation}=\;\frac{\mathrm{Patient}\;\mathrm{result}-\;\mathrm{Mea}n_{\mathrm{HC}}}{SD_{\mathrm{HC}}}$$

*Z*-scores >1.96 were considered significantly different compared with HCs. To evaluate the level of longitudinal change in patients, we marked results as significant if the difference between baseline and follow-up was >1 SD of the HC group. We chose this threshold as a conservative estimate of the methods’ variability because longitudinal MRI data in healthy subjects were not available. To check whether the longitudinal changes in patients are indeed larger than the physiological variability of the methods, comparable values from the literature were considered. Recent studies reported scan–rescan coefficients of variation (CV = deviation between consecutive measurements/mean of the measurements) for global myelin quantification with SyMRI in HC of ≤1.2% and region-based reproducibility of T_1_, T_2_, and PD in global WM of ≤1%.^5,24^ For ROI-derived DTI parameters in HC, longitudinal CV between 2 and 10% were reported.^25–27^

Additionally, we calculated *z*-deviation maps of the patients’ DTI and SyMRI scalars to provide whole-brain voxel-wise comparability between patients and HCs. For that, the mean and standard deviation maps of the HC groups were generated using fslmaths. For the individual patient maps in MNI space, *z*-maps for each of the DTI and SyMRI parameters were created using voxel-wise subtraction of the mean HC value and subsequent division through the standard deviation HC map value. As a result, the patient’s *z*-maps consisted of *z*-scores within each voxel, representing increases and decreases of patient values from normal references at a glance and independently from the individual parameter’s scale.

## Results

### Evolution of the patients’ clinical statuses and macroscopic MRI pathology between baseline and 6 months follow-up

The patients’ demographics and clinical statuses at 6 months follow-up are summarized in Table 1.

**Table 1:** Patients’ clinical status assessed at 6 months follow-up

	Age (years)	Disease duration (years)	Clinical status at 6 months follow-up		
			Visual function	Cognitive function	Motoric function
Index patient	30	1	Complete recovery of vision	Improvement	Ability to walk independently again
			P100(r): 108.8 ms^II^ (before 145.0 ms)^I^		
			P100(l): 126.3 ms^II^ (before 160.5 ms)^I^		
Patient 2	35	6	slight improvement of visual acuity and visually evoked potentials	Constant midgrade cognitive impairment	Stable condition of right-sided paraspastic
			P100(r): 103.0 ms^II^ (before 95.6 ms)^I^		
			P100(l): not measurable^II^ (before 99.7 ms)^I^		
Patient 3	30	7	improvement of visual acuity	Constant midgrade cognitive impairment	Stable condition of paraspastic
			P100(r): 110.0 ms^II^ (before 114.0 ms)^I^		
			P100(l): 112.0 ms^II^ (before 112.0 ms)^I^		

VEP was performed at baseline, 1 week after therapy initiation (I), and 6 months after therapy initiation (II).

The index patient showed a widespread symmetrical paraventricular leucoencephalopathy at baseline (Fig. 1 and Supplementary Fig. 1) covering occipital, frontal, and parietal WM, but mostly sparing the corpus callosum. At 6 months follow-up, the lesion volume had diminished considerably by 58% (Table 2). Compared with Patient 1, WM lesions of the other two patients were much smaller and were mainly located in occipital areas, showing less reduction at follow-up (Table 2, Supplementary Fig. 1).

**Table 2:** Whole brain, myelin, and lesion volumes for the three patients and the healthy control group

	WM (%ICV)	GM (%ICV)	CSF (%ICV)	NON (%ICV)	MVF (%ICV)	BPF (%ICV)	Lesion volume (mL)
**Pat1 BL**	24.0	39.0	32.0	5.0	6.0	68	48.45
**Pat1 FU**	26.7	47.2	24.2	2.0	6.6	75.8	28.68
**Pat2 BL**	28.1	39.1	30.5	2.3	5.2	69.5	10.16
**Pat2 FU**	25.4	42.8	29.7	2.0	5.7	70.3	8.87
**Pat3 BL**	26.4	44.1	27.7	1.8	5.8	72.3	6.8
**Pat3 FU**	25.0	46.8	26.7	1.4	6.3	73.3	4.4
**HC mean**	35	53	12	1	10	89	
**HC SD**	2.99	3.38	2.86	0.27	0.27	2.86	

Numerical results were extracted from the SyMRI Software.

BL, baseline; BPF, brain parenchymal fraction; CSF, cerebrospinal fluid; FU, follow-up; GM, grey matter; HC, healthy control; ICV, intracranial volume; MVF, myelin volume fraction; SD, standard deviation; WM, white matter.

All three patients exhibited reduced GM and WM volumes compared with the HC group at baseline, as well as a marked reduction in MVF across the whole brain (Table 2). From baseline to follow-up, myelin volumes, as well as brain parenchymal fraction (BPF) and GM volumes, increased.

### Index patient

#### Global brain myelin quantification

At treatment initiation, whole-brain MVF (as a percentage of ICV) of the index patient was considerably reduced in comparison with HCs (6 versus 10%), but recovered to a, nonetheless subnormal, level of 6.6% at 6 months follow-up (Table 2).

Similarly, RD quantification based on DTI, which served as a comparative method representing demyelination, showed a marked increase in RD in the index patient at baseline compared with HC (1.34 versus 0.79 × 10^–3^ mm^2^/s), and a convergence to normal values at follow-up (1.19 × 10^–3^ mm^2^/s; Table 3).

**Table 3:** DTI parameters for the index patient at baseline and follow-up and for the healthy controls

	FA			MD (mm^2^/s)			RD (mm^2^/s)			AD (mm^2^/s)		
	HC	Patient		HC	Patient		HC	Patient		HC	Patient	
ROI	Mean (CI)	BL mean	FU mean	Mean (CI) ×10^−3^	BL mean ×10^−3^	FU mean ×10^−3^	Mean (CI) ×10^−3^	BL mean ×10^−3^	FU mean ×10^−3^	Mean (CI) ×10^−3^	BL mean ×10^−3^	FU mean ×10^−3^
Whole_brain	0.21 (0.12–0.31)	0.15	0.17	0.89 (0.63–1.15)	**1.43**	**1.29** ^a^	0.79 (0.53–1.06)	**1.34**	**1.19** ^a^	1.09 (0.81–1.37)	**1.61**	**1.48** ^a^
Lesion	0.36 (0.24–0.49)	**0.21**	**0.24**	0.76 (0.64–0.87)	**1.21**	**1.07** ^a^	0.6 (0.47–0.74)	**1.08**	**0.94** ^a^	1.06 (0.88–1.24)	**1.47**	**1.33**
Corpus_callosum	0.54 (0.44–0.64)	**0.38**	**0.37**	0.96 (0.76–1.17)	**1.27**	1.1^a^	0.65 (0.45–0.86)	**1.02**	**0.88** ^a^	1.59 (1.31–1.87)	1.78	1.56
Sup_corona_radiata_L	0.42 (0.32–0.53)	0.32	0.41	0.72 (0.56–0.88)	**1.11**	**0.89** ^a^	0.54 (0.39–0.7)	**0.92**	0.68^a^	1.07 (0.86–1.29)	**1.48**	**1.3** ^a^
Sup_corona_radiata_R	0.43 (0.31–0.54)	0.32	0.34	0.7 (0.54–0.87)	**1.11**	**0.99** ^a^	0.53 (0.37–0.69)	**0.91**	**0.81** ^a^	1.05 (0.83–1.28)	**1.5**	**1.36** ^a^
Posterior_thalamic_rad_L	0.45 (0.35–0.55)	**0.22**	**0.29** ^a^	0.95 (0.71–1.19)	**1.64**	**1.34** ^a^	0.71 (0.48–0.94)	**1.45**	**1.15** ^a^	1.44 (1.1–1.78)	**2.0**	1.73^a^
Posterior_thalamic_rad_R	0.48 (0.37–0.59)	**0.21**	**0.22**	0.87 (0.69–1.04)	**1.59**	**1.63**	0.62 (0.44–0.8)	**1.42**	**1.46**	1.36 (1.08–1.64)	**1.93**	**1.97**
Post_limb_internal_capsule_L	0.55 (0.41–0.69)	0.53	0.58	0.7 (0.49–0.9)	0.82	0.77	0.46 (0.28–0.64)	0.56	0.48	1.17 (0.87–1.47)	1.34	1.35
Post_limb_internal_capsule_R	0.56 (0.4–0.73)	0.56	0.48	0.65 (0.43–0.87)	0.74	0.78	0.42 (0.22–0.62)	0.48	0.55	1.11 (0.79–1.43)	1.27	1.24
Cerebral_peduncle_L	0.57 (0.42–0.71)	0.5	0.45	0.77 (0.53–0101)	0.79	0.87	0.5 (0.29–0.71)	0.56	0.65	1.31 (0.94–1.68)	1.24	1.33
Cerebral_peduncle_R	0.58 (0.41–0.74)	0.64	0.47	0.76 (0.48–1.04)	0.65	0.73	0.5 (0.24–0.75)	0.4	0.52	1.3 (0.89–1.7)	1.16	1.14
Corticospinal_tract_L	0.41 (0.29–0.52)	0.38	0.4	0.75 (0.49–1.01)	0.86	0.75	0.58 (0.32–0.84)	0.7	0.58	1.09 (0.78–1.4)	1.18	1.07
Corticospinal_tract_R	0.42 (0.29–0.55)	0.4	0.39	0.73 (0.45–1.0)	0.9	0.81	0.55 (0.28–0.82)	0.73	0.63	1.08 (0.74–1.42)	1.24	1.17

CI, mean ± 1.96 × SD. Patient’s mean values outside CI are reported in bold.

AD, axial diffusivity; BL, baseline; CI, confidence interval; HC, healthy controls; FA, fractional anisotropy; MD, mean diffusivity; FU, follow-up; RD, radial diffusivity; ROI, region of interest; SD, standard deviation.

^a^ Marked differences between baseline and follow-up (>1 SD of HC group).

#### Lesion analysis

The comparison between our patients and HCs showed lower mean MVF, R1, and R2, and higher mean PD values within the WM lesion area in patients. From baseline to follow-up, all the index patient’s parameters within the baseline lesion area recovered (changes >1 SD of HC for R1 and R2), consistent with the patient’s clinical improvement, but did not reach the corresponding HC mean values (Table 4).

**Table 4:** Mean SyMRI values within the lesion and regions of interest for the index patient and the healthy control group

ROI	MVF (%)			PD (pu)			R1(1/s)			R2 (1/s)		
	HC	Patient		HC	Patient		HC	Patient		HC	Patient	
	Mean (CI)	BL mean	FU mean	Mean (CI)	BL mean	FU mean	Mean (CI)	BL mean	FU mean	Mean (CI)	BL mean	FU mean
Lesion	25.38 (17.75–33)	**12.7**	**14.41**	69.13 (63.75–74.51)	**78.91**	**76.78**	1.43 (1.22–1.64)	**1.03**	**1.14** ^a^	12.87 (11.8–13.94)	**8.08**	**9.88** ^a^
Corpus_callosum	22.55 (19.93–25.17)	22.27	22.3	72.39 (70.25–74.53)	**75.02**	73.56	1.31 (1.22–1.41)	**1.19**	1.25^a^	12.47 (11.93–13)	**11.17**	**11.38**
Sup_corona_radiata_L	27.46 (23.86–31.05)	**19.01**	**21.12** ^a^	67.74 (65.08–70.4)	**73.59**	**72.15** ^a^	1.46 (1.34–1.59)	**1.21**	**1.27**	12.67 (11.87–13.47)	**9.82**	**10.66** ^a^
Sup_corona_radiata_R	26.79 (22.89–30.69)	**18.2**	**22.52** ^a^	68.18 (65.43–70.92)	**74.31**	**71.17** ^a^	1.45 (1.32–1.58)	**1.16**	**1.25** ^a^	12.64 (11.78–13.49)	**9.29**	**10.17** ^a^
Post_thalamic_rad_L	31.81 (30–33.62)	**12.12**	**18.07** ^a^	66.04 (62.42–69.66)	**79.86**	**74.92** ^a^	1.61 (1.44–1.78)	**0.98**	**1.24** ^a^	13.22 (12.17–14.27)	**7.24**	**9.59** ^a^
Post_thalamic_rad_R	31.53 (28.65–34.4)	**11.52**	**16.15** ^a^	65.2 (62.34–68.06)	**79.83**	**76.06** ^a^	1.63 (1.5–1.77)	**0.95**	**1.17** ^a^	13.6 (12.83–14.38)	**7.33**	**9.72** ^a^
Post_limb_int_capsule_L	29.07 (25.02–33.13)	26.69	27.05	65.48 (63.43–67.54)	**68.1**	**67.82**	1.42 (1.31–1.52)	1.41	1.39	13.11 (12.48–13.74)	12.68	**12.15**
Post_limb_int_capsule_R	28.34 (24.48–32.2)	25.48	26.16	66.2 (64.24–68.16)	**68.98**	**68.53**	1.41 (1.32–1.51)	1.42	1.39	13.16 (12.5–13.83)	12.62	**11.99**
Cerebral_peduncle_L	22.29 (18.9–25.68)	22.88	20.3	70.16 (67.66–72.65)	71.37	72.3	1.37 (1.27–1.47)	1.41	1.32	13.28 (12.46–14.1)	13.68	12.68
Cerebral_peduncle_R	20.56 (15.45–25.67)	20.76	20.23	71.53 (68.42–74.63)	72.19	72.27	1.36 (1.25–1.47)	1.38	1.32	13.46 (12.59–14.32)	13.99	**12.55**
Corticospinal_tract_L	23.35 (18.35–28.35)	**28.81**	23.71	69.83 (66.2–73.46)	**65.99**	70.44	1.34 (1.25–1.42)	1.42	1.36	12.6 (11.8–13.4)	12.6	12.96
Corticospinal_tract_R	22.98 (18.49–27.47)	26.71	21.7	70.06 (67.04–73.07)	67.71	71.54	1.34 (1.25–1.43)	1.38	1.4	12.64 (11.71–13.56)	13.16	13.6

CI = mean ± 1.96 × SD. Patient’s mean values outside CI are reported in bold.

^a^ Marked differences between baseline and follow-up (>1 SD of HC group).

BL, baseline; CI, confidence interval; FU, follow-up; HC, healthy controls; PD, proton density; MVF, myelin volume fraction; ROI, region of interest; SD, standard deviation.

DTI quantification within the lesion showed significant differences of the patient’s DTI parameters compared with controls in the lesion area. Thus, mean FA values were lower, and MD, RD, and AD were higher on average (Table 3). Longitudinally, a recovery was observed for MD and RD.

#### ROI-based analysis

**Figure 3:**
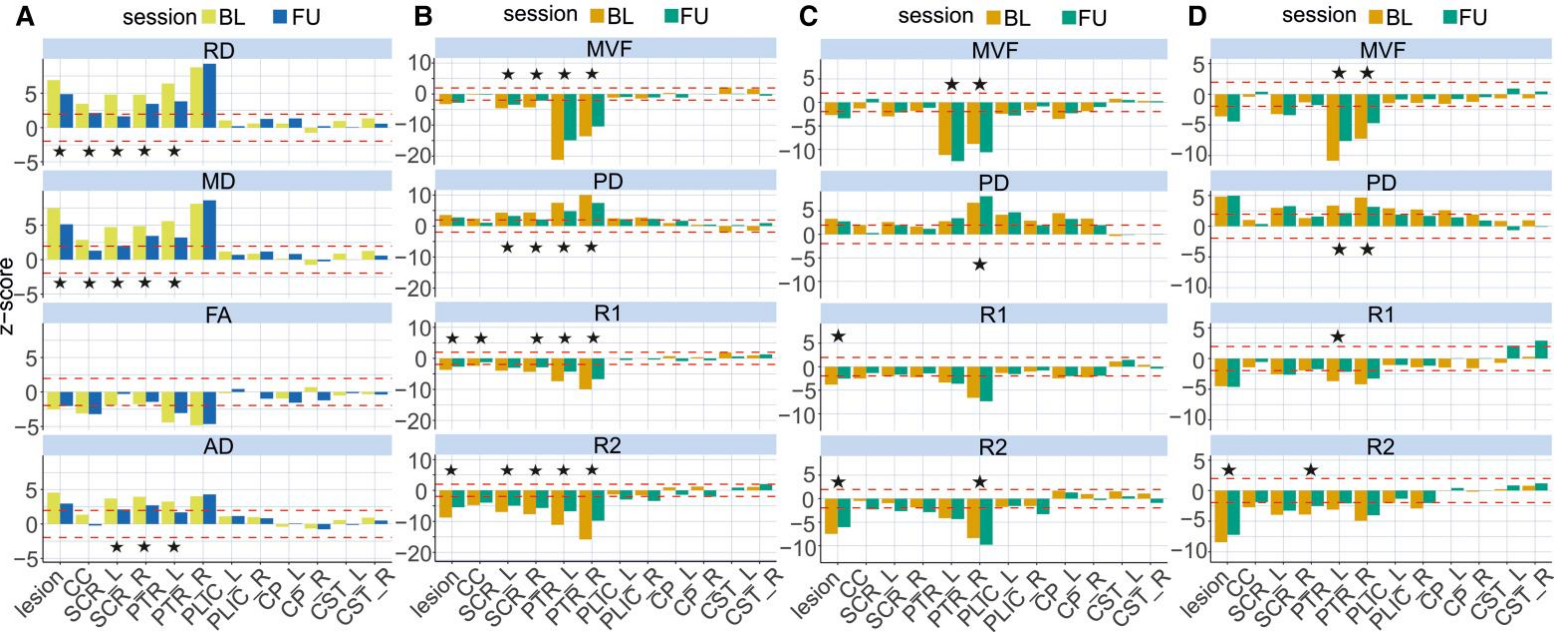
**Regional *z*-scores.** Mean regional *z*-scores for synthetic and DTI parameters at baseline (BL) and follow-up (FU). The red horizontal line depicts the threshold of 1.96. Stars highlight marked differences between baseline and follow-up (>1 SD of HC group). (**A**) DTI *z*-scores of the index patient. (**B**) Synthetic MRI *z*-scores of the index patient. (**C**) Synthetic MRI *z*-scores of Patient 2. (**D**) Synthetic MRI *z*-scores of Patient 3. CC, corpus callosum; CP, cerebral peduncle; CST, cortical spinal tract; L, left; PLIC, posterior limb of internal capsule; PTR, posterior thalamic radiation; R, right; SCR, superior corona radiata.

Table 4 summarizes the mean SyMRI parameter values for the index patient and the control group within several ROI, which were selected according to the patient’s leading symptoms (impairment of vision and motoric functions). In addition, Fig. 3A and B depicts the deviations of the index patient’s results from the control group expressed as *z*-scores. We observed lower mean MVF, R1, and R2, and higher mean PD values within the posterior thalamic radiation (including optic radiation) and the superior corona radiata at baseline, which constitute regions most affected by the leucoencephalopathic lesions. In these regions, SyMRI results showed a marked recovery during follow-up (changes >1 SD of HC). In contrast, in other ROIs that are part of the major descending motor tracts (posterior limb of internal capsule, CST, and cerebral peduncles), only slight deviations from normal references were observed. Thus, the posterior limb of internal capsule showed higher mean PD values bilaterally at both time points. We observed no significant MVF alteration in the corpus callosum, while PD, R1, and R2 were significantly altered at baseline and R1 returned to normal levels at follow-up.

In general, the highest absolute *z*-scores, exceeding 20, were observed for MVF (Fig. 3B), but the *z*-scores that exceeded the threshold of 1.96 were restricted to ROIs that had the largest overlap with the lesion area, namely the bilateral posterior thalamic radiation (including optic radiation) and the superior corona radiata.

In accordance with our results from SyMRI, we observed significant changes of the index patient’s DTI parameters MD and RD compared with controls in the posterior thalamic radiation (including optic radiation) and superior corona radiata. Thus, mean MD, RD, and AD were higher in these regions at baseline and considerably lower during follow-up, except for the right posterior thalamic radiation (including optic radiation; Table 3). Mean FA values were significantly lower within the bilateral posterior thalamic radiation (including optic radiation) at both baseline and follow-up. In the corpus callosum, RD, MD, and FA were significantly altered at baseline, and RD and MD recovered to normal levels.

Overall, regional *z*-deviations were most pronounced for MD and RD, both of which showed similar *z*-scores of around five to eight in the posterior thalamic radiation (including optic radiation) and the superior corona radiata (Fig. 3A).

The thresholds of 1 SD of the results in the HC group, which we chose to assess the magnitude of longitudinal changes in patients, corresponded on average (across all ROI) to a CV of 7.7% for MVF, 2.0% for PD, 4.1% for R1, 3.3% for R2, 14.0% for FA, 14.0% for MD, 18.8% for RD, and 12.4% for AD. Although these cross-sectional thresholds were not identical to a longitudinal repeatability measure, the thresholds were clearly above the longitudinal variability found in global WM in healthy individuals reported in literature (for SyMRI: CV <1.2% in MVF, PD, R1, and R2, for DTI: longitudinal CV between 2 and 10% in FA, MD, RD, and AD, see Statistics section).^5,24–27^

#### Voxel-based analysis using *z*-maps

**Figure 4:**
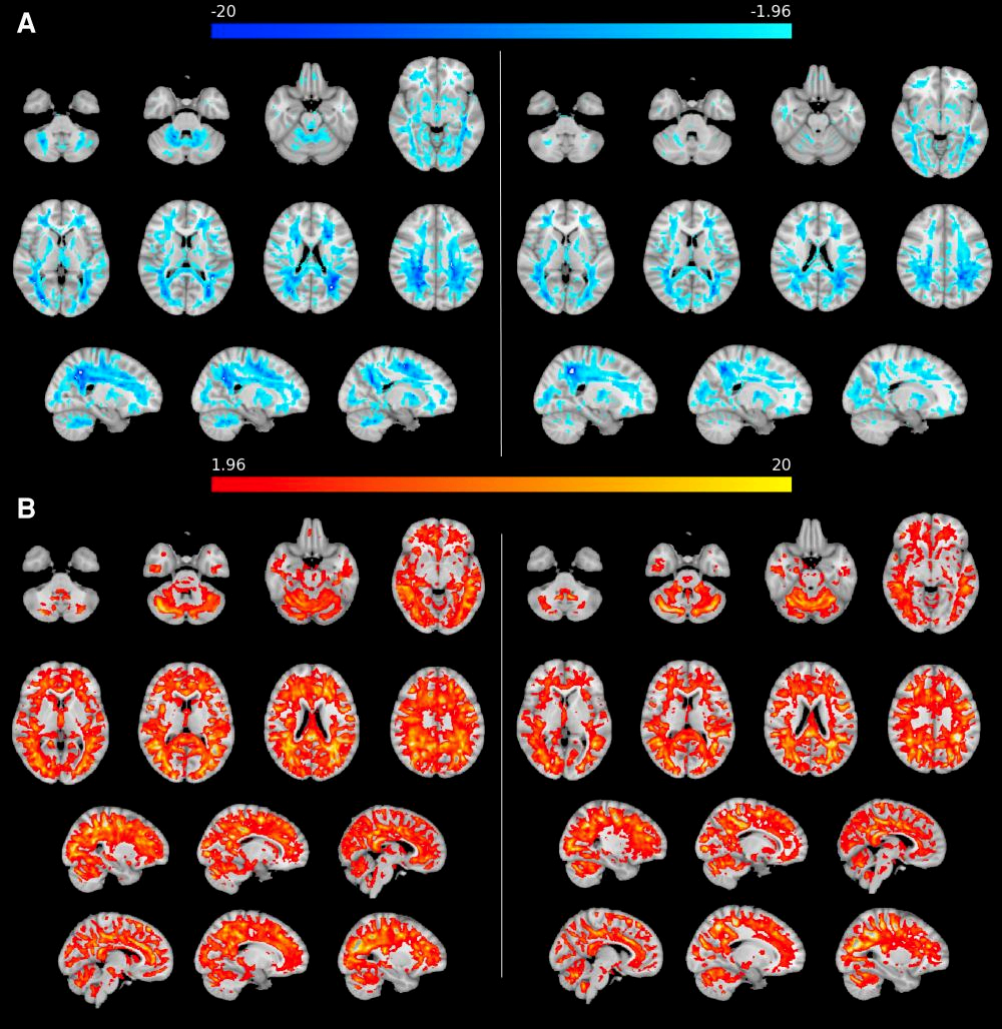
**Myelin and RD *z*-maps.** (**A**) *Z*-deviation map for voxel-wise myelin volume fraction showing considerable decreases of the index patient’s myelin from healthy controls. Left: baseline, right: follow-up. The *z*-map is depicted as blue overlay on the MNI152 T1 template and displays areas in which the *z*-deviation exceeds 1.96. (**B**) *Z*-deviation map for radial diffusivity (RD) showing considerable increases of the index patient’s RD from healthy controls. Left: baseline, right: follow-up. The *z*-map is depicted as orange overlay on the MNI152 T1 template and displays areas in which the *z*-deviation exceeds 1.96.

The *z*-deviation maps represented the patient’s voxel-based deviation from HCs across the whole brain. Our *z*-maps illustrated the improvement of MVF from baseline to follow-up as decreasing deviation from HC, expressed by increasing negative *z*-scores. They represented voxel-wise, whole-brain changes, beyond the preselected atlas ROIs. In addition to high *z*-scores in the lesion area, we observed high deviations in the precentral and postcentral gyrus, in frontal regions, in the splenium of the corpus callosum, and in other parts of the cerebellum which were less pronounced in the follow-up images (Fig. 4A).

In line with the MVF results, *z*-deviation maps for RD showed a marked decline of *z*-values at follow-up, which were even halved in some regions, such as the superior and posterior corona radiata (Fig. 4B). Overall, high RD *z*-deviations were found in the same regions as in MVF but seemed to be distributed more diffusely throughout the brain.

### Quantitative MRI in other patients with MTHFR deficiency (Patients 2 and 3)

Patients 2 and 3 showed lower mean MVF, R1, and R2 as well as higher mean PD values within the WM lesion area compared with the control group (Fig. 3C and D). For the two patients, who showed only mild symptom improvement between baseline and 6 months follow-up, mean SyMRI values only slightly differed between baseline and follow-up (Fig. 3C and D). Again, the highest absolute *z*-scores were observed for MVF, which reached *z*-scores of around −10 in the posterior thalamic radiation (including optic radiation). Compared with the index patient, absolute *z*-scores for Patients 2 and 3 were lower for most of the ROIs, but showed a similar pattern of most affected regions, namely the posterior thalamic radiation (including optic radiation) and the superior corona radiata.

## Discussion

Increasing efforts are being made to improve MRI acquisition methods for noninvasive estimation of myelin content of human brain tissue, with the intention of monitoring myelin longitudinally and assessing treatment efficacy.^28^ Especially in demyelinating diseases, such as MS, various MRI techniques are used to study pathological changes in the brain leading to demyelination. The need for these techniques is driven further by emerging remyelinating pharmacotherapies.^29^ Nevertheless, myelin quantification often requires lengthy multimodal MRI protocols and sophisticated data processing techniques, which is why they are rarely used in routine clinical practice. In our study, we used SyMRI, a fast, single-sequence multiparametric quantitative MRI method which automatically quantifies the MVF through simultaneous measurements of relaxometry parameters (R1 and R2 relaxation rates and PD).^14^ Based on the advanced neuroimaging evaluation of a patient with MTHFR deficiency as a characteristic model of primary rapid demyelination with following treatment-induced remyelination, we measured myelin evolution longitudinally using SyMRI, compared with established DTI methods. We included three patients with MTHFR deficiency to monitor rapid disease-related demyelination and therapy-induced remyelination. We analysed myelin correlating parameters at global, region-based, and voxel-based levels, comparing individual patient data to groups of matched HCs.

### Improvement of the patients’ clinical status between baseline and 6 months follow-up

The index-patient (Patient 1) experienced significant clinical improvements of visual, cognitive, and motor functions following high-dose betaine and supporting methionine treatment. The baseline to 6 months follow-up recovery was reflected by quantitative markers, such as a normalisation of substantially prolonged P100 latency in visually evoked potentials and diminishing leucoencephalopathy in conventional T_2_-FLAIR-weighted MRI. In Patients 2 and 3 with MTHRF deficiency, visual acuity improved slightly (Patient 3), while cognitive and motor functions remained constant. During follow-up, their leucoencephalopathy on MRI only slightly decreased.

### Global brain quantitative MRI

Whole-brain quantification from SyMRI indicated reduced myelin fractions in all three patients compared with HCs. MVF increased substantially between baseline and follow-up in the early treated index Patient 1 and, less pronounced, in Patients 2 and 3 with longer disease duration before treatment initiation. Accordingly, for Patient 1, the DTI-derived RD of the whole-brain, regarded as a different myelin marker, partly recovered during follow-up from abnormal levels at baseline. These findings corroborate that automatic global quantification of MVF with SyMRI is suitable for detecting demyelination and remyelination at global levels. This is consistent with previous findings that SyMRI is useful for showing myelin-related abnormalities in other diseases, like Sturge–Weber syndrome.^30^ Furthermore, global brain volume fractions of WM, GM, and BPF of the patients were reduced compared with HCs and showed marked recovery at follow-up, supporting that the SyMRI technique with automatically obtained global parameters was suitable to measure global changes of brain volumes and myelin content.

### ROI-based myelin quantification and *z*-maps of the index patient

In Patient 1, quantitative comparisons between SyMRI and DTI metrics were made within the lesion area affected by leucoencephalopathy, in atlas-based regions associated with leading symptoms (optic radiation, motoric tracts, corona radiata and corpus callosum for visual, motoric and cognitive impairment), and in voxel-wise *z*-deviation maps. Overall, MVF was the MRI parameter, which showed the most pronounced differences between patients and HC, supported by DTI metrics showing the greatest *z*-deviations of RD in Patient 1.

Within the lesion area, all mean SyMRI values and diffusions metrics of the patient differed significantly from HCs, both at baseline and at 6 months follow-up. Herein we observed lower MVF, R1, R2, and FA and higher PD, RD, MD, and AD measures. Recently, similar concurring changes of SyMRI and DTI parameters have been detected in cross-sectional studies of MS WM abnormalities, where reduced MVF, R1, R2, and increased PD corresponded to increased RD, MD, AD, and reduced FA in MS lesions compared with NAWM and HC.^3^ Another study indicated a higher sensitivity of the MVF compared with R1, R2, and PD for capturing WM damage in MS.^31^ Hagiwara *et al*.^3,31^ could not demonstrate significant differences of R1, R2, and PD between patients with MS and HCs compared with MVF from TBSS analysis. This confirmed previous findings showing higher sensitivity of MVF compared with R1, R2, and PD for revealing WM damage in MS. Longitudinally, the SyMRI and DTI metrics had both attained incomplete recoveries at follow-up in the present study. Remyelination within the lesion was reflected by MVF increasing from 12.7% at baseline to 14.4% at follow-up, confirmed by changes in diffusion metrics.

For Patient 1, ROI-based analysis demonstrated largely corresponding SyMRI and DTI changes in the large supratentorial WM tracts that were affected by visible leucoencephalopathy, such as the corona radiata and the posterior thalamic radiation (including optic radiation). Consistently, highest absolute *z*-scores, reflecting the highest deviation from HC values, were observed for MVF in the left and right posterior thalamic radiation (including optic radiation) at baseline and reducing at follow-up, which could correspond to remyelination. MVF changes were consistent with *z*-scores of PD, R1, and R2 at baseline and follow-up. At baseline, the DTI indices within this region conformed to MVF changes in both hemispheres but did not indicate restitution in the right hemisphere at follow-up. This could be due to the accentuated cerebral volume reduction in this region (Fig. 1). Diffusion metrics could also be influenced by local atrophy in which neurodegeneration and demyelination are closely associated.

Interestingly, MVF reduction was not detectable in the corpus callosum of the index patient. The unaffected MVF level in the corpus callosum of the index patient could be due to the genetic nature of MTHFR deficiency as a systemic metabolic disease with diffuse affection and demyelination of the WM, which is not prone to neuroinflammation and degeneration. In contrast, myelin measures of the corpus callosum in MS showed reduced myelin water fraction compared with controls.^32^ However, we observed marked alterations of DTI metrics in the corpus callosum for the index patient at baseline compared with HC, mainly of RD, MD, and FA. In contrast, AD, which together with RD attributes to demyelination, was unchanged. Limitations of conventional diffusion metrics, such as the ones used here, to discriminate between axonal injury and demyelination have been described for the corpus callosum, characterized by densely packed axons.^33,34^ We hypothesized that even in our index patient DTI would be limited in its ability to characterize microstructural changes of the corpus callosum. Although Patient 1 was severely physically disabled by tetraparesis and ataxia at baseline, we found no indications of major myelin alterations in the ROIs representing descending motoric pathways (posterior limb of the internal capsule, cerebral peduncles, and corticospinal tracts); neither in MVF, nor in RD. However, the voxel-wise *z*-deviation maps of MVF and RD indicated widespread demyelination of other brain structures in this patient. Specifically, the cerebellum and WM of the pre- and postcentral gyri showed marked *z*-deviations at baseline, which decreased at follow-up. Demyelination in areas closely involved in motoric function and regulation, especially in the cerebellum, could constitute structural correlates of observed motoric symptoms. Overall, *z*-deviation maps for RD demonstrated marked and widespread RD alterations in supra-and infratentorial regions, mostly excluding deep GM regions at baseline and a decline of these *z*-deviations at follow-up. Compared with MVF *z*-deviations maps, RD deviations were distributed more diffusely, which may reflect a coexistence of demyelination and axonal degeneration. This is in line with findings of widespread abnormalities in the WM of patients with MS reflected by RD changes,^35^ indicating a more specific detection of myelin changes by MVF. The extent of myelin deviation compared with HCs and the more focused regional affection may support the hypothesis that MVF is suitable for detecting myelin changes in the index patient more specifically than RD. However, MVF and RD generally showed consistent changes between Patient 1 and the HC group supporting our hypotheses that MVF derived by SyMRI is applicable for estimating brain myelin content. Our findings regarding MVF support recent histopathological post-mortem analyses validating myelin quantification using SyMRI.^5,36^ Even in MS, the relationship between RD and MVF measured by SyMRI is in agreement.^4^ Since RD was described less specific to myelin integrity,^33^ the comparison with MVF may lead to additional information about demyelination and remyelination.

### Disease-specific conclusions regarding the index patient

For Patient 1, SyMRI- and DTI-derived metrics revealed indications of demyelination at baseline, extending far beyond the leucoencephalopathic lesions visible on T_2_-FLAIR MRI. Therein we detected cerebellar involvement and affection of the pre- and postcentral motoric areas, which may have contributed to the patient’s motoric symptom spectrum. Marked demyelination of the optic radiation may correspond to the loss of vision at baseline. Furthermore, we found involvement of the frontal lobes and the splenium of the corpus callosum. During follow-up, both MVF and RD changes suggest remyelination on the global and local level, presumably corresponding to the patient’s substantial clinical recovery of vision and motoric functions. The estimation of remyelination of the visual pathways from baseline to follow-up in the index patient was further supported by the congruent improvement of P100 latency measured by visual-evoked potentials as a marker of demyelination as well as remyelination.^37^

### Comparison with other patients with MTHFR deficiency

The SyMRI results of Patients 2 and 3 with MTHFR deficiency were in line with the values of the index patient. Their global brain myelin content was reduced in comparison with HCs and showed a slight recovery at 6 months follow-up. The ROI-based analysis confirmed the global myelin quantification. We observed pronounced *z*-deviations in the in WM lesion area and in the posterior thalamic radiation (including optic radiation) of Patients 2 and 3. During follow-up, a decline of the *z*-deviation, indicating remyelination, was found in the posterior thalamic radiation (including optic radiation) of Patient 3, in congruence with clinical improvement of vision in Patient 3. In Patients 2 and 3, significant short-term remyelination was not expected, as, in contrast with Patient 1, the treatment initiation was significantly delayed (6 and 7 years after manifestation).

Based on the results of the three patients, we assume that whole-brain myelin measurement is suitable for estimating global myelin content to monitor myelin dynamics and evaluate treatment aiming at short-term remyelination. Regional MVF analysis and comparison with diffusion metrics confirm the method’s value and consistency with established markers of demyelination in the index patient. The good agreement between both methods supports a reasonable estimation of the myelin content, whereby myelin estimation by SyMRI is a time-saving and user-friendly method, which additionally provides quantitative MRI measures like GM and WM volume as well as BPF.^38^ In contrast, DTI analysis requires technically challenging pre- and postprocessing methods, while SyMRI provides automatic brain segmentation and myelin measurement with a dedicated postprocessing software time of <1 min.^2^

### Pathophysiological processes and remyelination in MTHFR

In most studies the pathophysiological background of de- and remyelination in MTHFR deficiency has been inferred from laboratory studies or from qualitative changes on MRI, whereas autopsy studies or quantitative MR investigations in this disease are very rare. MTHFR deficiency causes elevated plasma levels of homocysteine and decreased blood methionine levels by insufficient remethylation of homocysteine back to methionine, resulting in demyelination. This has been proved in autopsy studies from patients with MTHFR deficiency.^11^ In addition, a decrease of brain demyelinated involvement following betaine administration could be verified in conventional MRI and MR spectroscopy.^12^ In a recent validation study of rapid magnetic resonance myelin imaging in MS, the association of myelin estimation by SyMRI with myelin stains was numerically higher than with axonal stains, so it was assumed that the sequence reflects myelin content rather than axons.^5^ Although it cannot principally be assumed that the pathophysiological processes involved in demyelination are the identical in MS and MTHFR deficiency, there are nevertheless some similarities between the two diseases that make a model transfer plausible. In MS, elevated homocysteine levels in the absence of MTHFR deficiency as well as an association with clinical disability could be shown by several studies.^39–41^ The cause is assumed to be an excitotoxicity induced by hyperhomocysteinaemia as well as a reduced availability of methionine as methyl group donor, which result in hypomethylation of myelin basic protein and loss of stability of myelin structures.^42,43^ Based on the specific pathophysiological information about MTHFR deficiency and the analogy with myelin quantification in MS, we conclude that the observation of relevant demyelination and therapeutically induced remyelination in MTHFR deficiency is feasible. This may allow us to suppose MTHFR as a disease model for evaluation of myelin measures in short-term course at an individual patient level. Thus, it can be hypothesized that myelin quantification by SyMRI allows the assessment of de- and remyelination in supratentorial WM. Our results, which showed a concordant but in magnitude larger deviation of MVF than DTI parameters (highest absolute *z*-scores were observed for MVF), support this hypothesis. Remyelination was further reflected in our index patient by marked improvement of P-100 latency of visual-evoked potentials (right: 108.8 versus 145 ms before; left: 126.3 versus 160.5 ms before), which was accompanied by a significant recovery of visus.^13^

### Limitations

This explorative study is not without limitations. The number of patients is limited due to the rarity of this genetic disease. Additionally, the disease should serve as a model disease with rapid remyelination after treatment initiation to monitor short-term myelin changes. However, due to the small sample size, generalization to other demyelinating diseases is limited and our findings should be confirmed in larger samples of other diseases. Furthermore, the quantitative MRI results were not validated at a histopathological level. HCs were matched to Patients 2 and 3 for age, but not for sex, and we used different HC groups for SyMRI and DTI analysis. The slightly higher age in the 1.5 T control group might lead to a physiological bias in the MRI measures, for example, by a decrease of brain volumes or increase of relaxation times and thus may lead to an underestimation of the differences between patients and HCs compared with the DTI control group. This can also influence the analyses of *z*-scores and *z*-deviation maps. Still, we assume that these effects will be small compared with the processes we observed in the patients.

Another limitation is the lack of scan–rescan studies in healthy subjects, which would provide information about the reproducibility of the SyMRI and DTI result parameters over time. However, comparisons with published literature have shown that the cut-off values we chose for abnormal changes in both SyMRI and DTI parameters were clearly more restrictive than the reported scan–rescan reproducibility of global WM in healthy subjects. Even if we have to assume that the scan–rescan variability in smaller ROIs, such as the lesions in the MTHFR-deficient patients, might actually be larger (by about a factor of about 2^44^), the threshold values of 1 SD of the control group that we chose for comparison would still be suitable for assessing the relevance of the individual longitudinal changes in patients.

There are some further methodological limitations. Since different modalities with varying resolutions and processing methods were used, comparability of region-based analyses between these modalities is restricted. Both DTI and SyMRI images were registered to MNI space, but different methods were necessary to achieve the best alignment results. Moreover, diffusion images were corrected for distortion using nonlinear registration to structural T_1_ images, due to lacking acquisition of transverse diffusion scans with different phase-encoding directions.

A further limitation is the limited specificity of MVF as well as of DTI measures related to the differentiation of myelin and axonal pathology. Although it is assumed, based on the histopathological validation, that myelin estimation by SyMRI reflects the myelin content rather than the axons, it is acknowledged that there is a colocalization of myelin around the axons as well as the concomitant processes of demyelination and axonal loss.

### Conclusion

Based on this advanced neuroimaging evaluation by quantitative MRI techniques, we conclude that it can be assumed that SyMRI-based myelin estimation is an appropriate measure to estimate short-term myelin changes in diseases of the central nervous system, especially remyelination, even on individual patient levels. Prospectively, this time-saving and user-friendly tool may be suitable for monitoring myelin changes, even in the context of remyelinating therapy response in MS.

## Supplementary Material

fcac172_Supplementary_Data

## Data Availability

For data protection reasons, the data collected in this study cannot be made publicly available, but anonymized data of the control groups used for this study will be made available from the corresponding author on reasonable request. The MRI data of the patients cannot be provided for data protection reasons.

## Abbreviations

AD =: axial diffusivity
BPF =: brain parenchymal fraction
DTI =: diffusion tensor imaging
FA =: fractional anisotropy
GM =: grey matter
FLAIR =: fluid attenuated inversion recovery
HC =: healthy control
ICV =: intracranial volume
MD =: mean diffusivity
MTHFR =: methylenetetrahydrofolate reductase
MVF =: myelin volume fraction
NAWM =: normal appearing white matter
PD =: proton density
RD =: radial diffusivity
ROI =: region of interest
SyMRI =: synthetic MRI
TBSS =: tractography-based spatial statistics
WM =: white matter.
