# Supplementary material for: Quantification of individual remyelination during short-term disease course by synthetic magnetic resonance imaging
Source: Brain Commun. 2022 Jun 28;4(4):fcac172. doi: 10.1093/braincomms/fcac172 (PMC9351729; doi:10.1093/braincomms/fcac172)
Supplement: fcac172_Supplementary_Data [file fcac172_supplementary_data.pdf]

Supplementary material

Supplementary Figure 1: FLAIR images from synthetic MRI for the three patients.

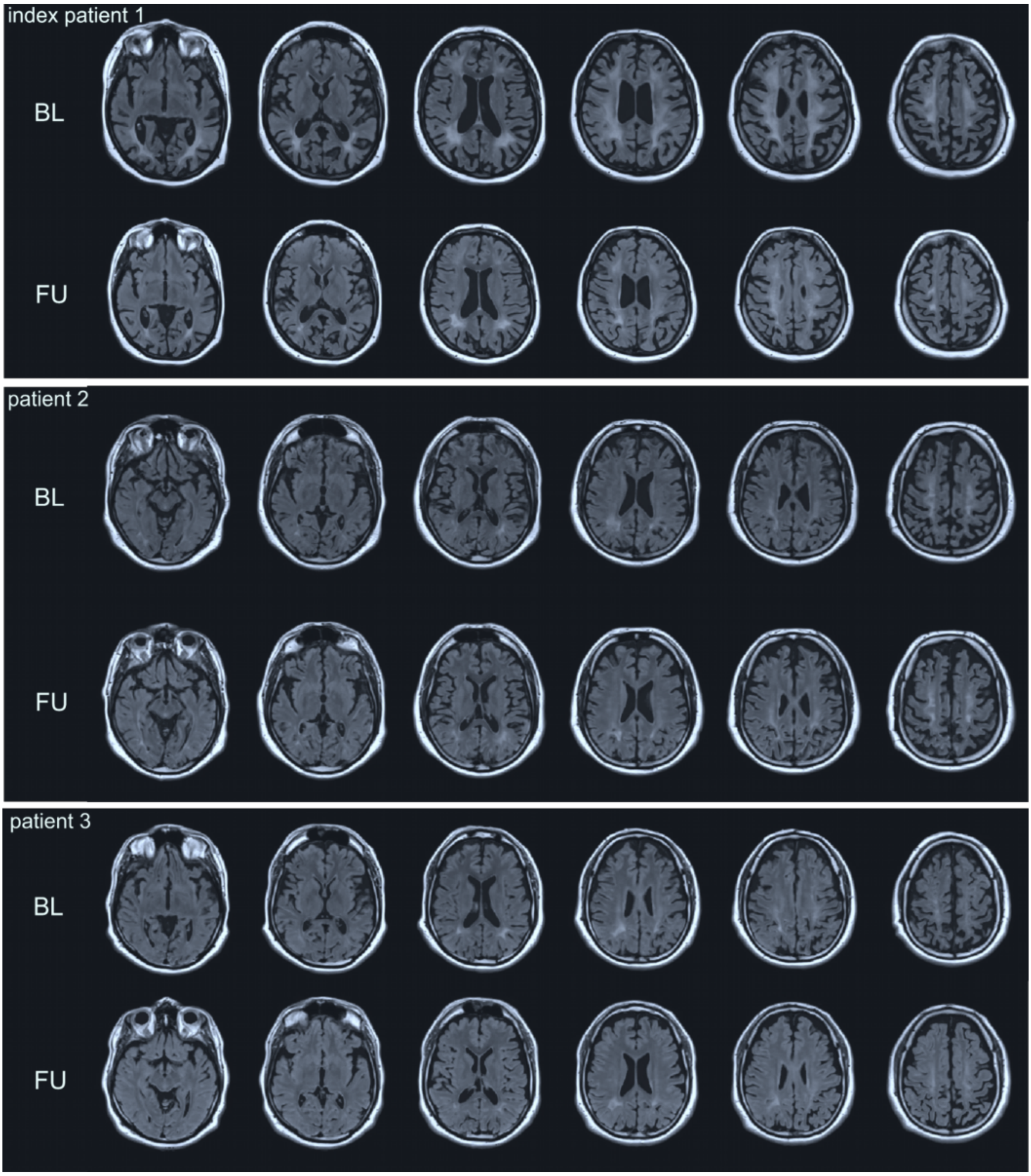

Upper rows: index patient; middle rows: patient 2; lower rows: patient 3. Baseline (BL) and Follow-up (FU) scans are displayed below one another for each patient. Z-axis range: 57-114.

Supplementary Figure 2: Schematic description of the processing of diffusion images.

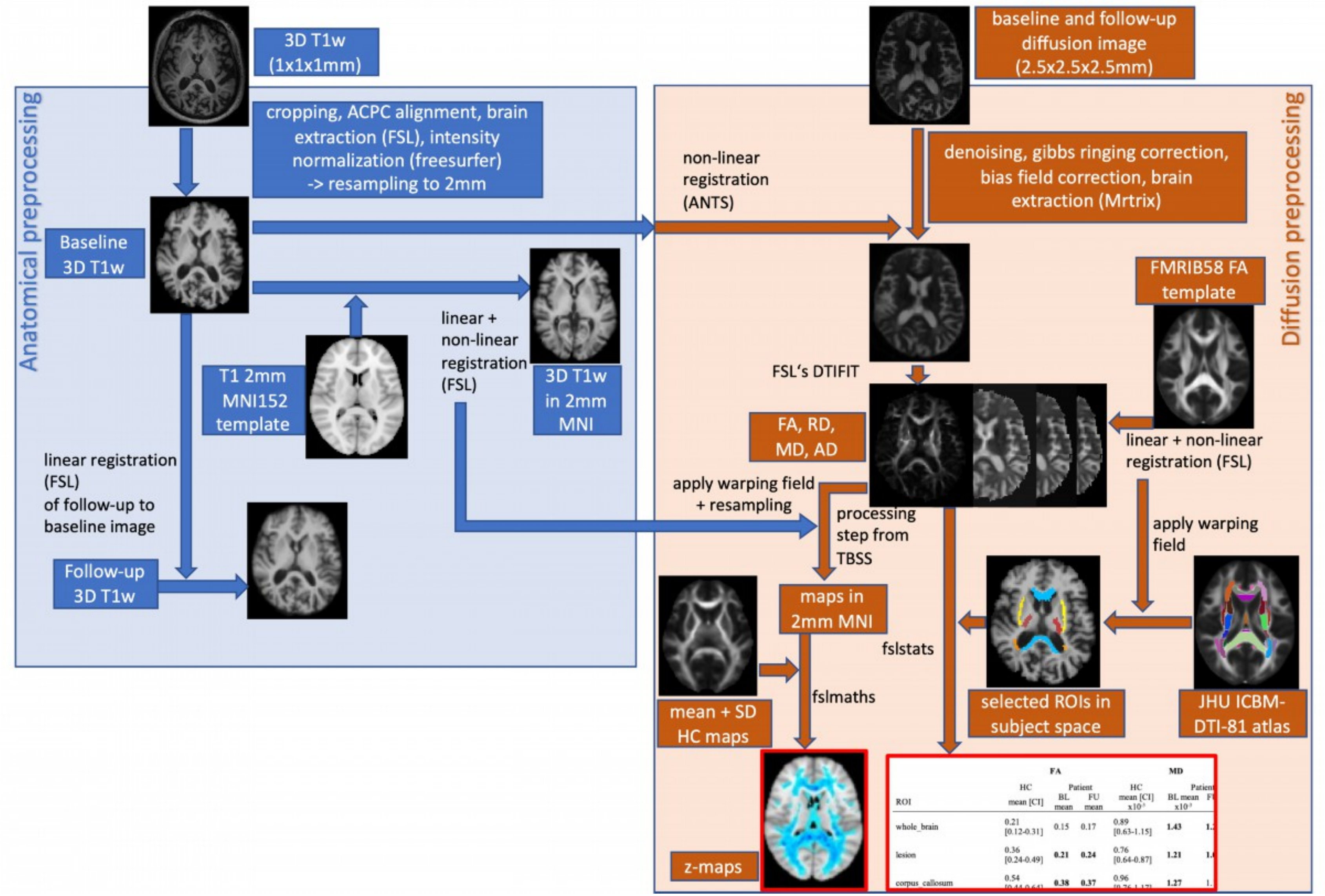

Supplementary Figure 3: Schematic description of the processing of Synthetic MRI data.

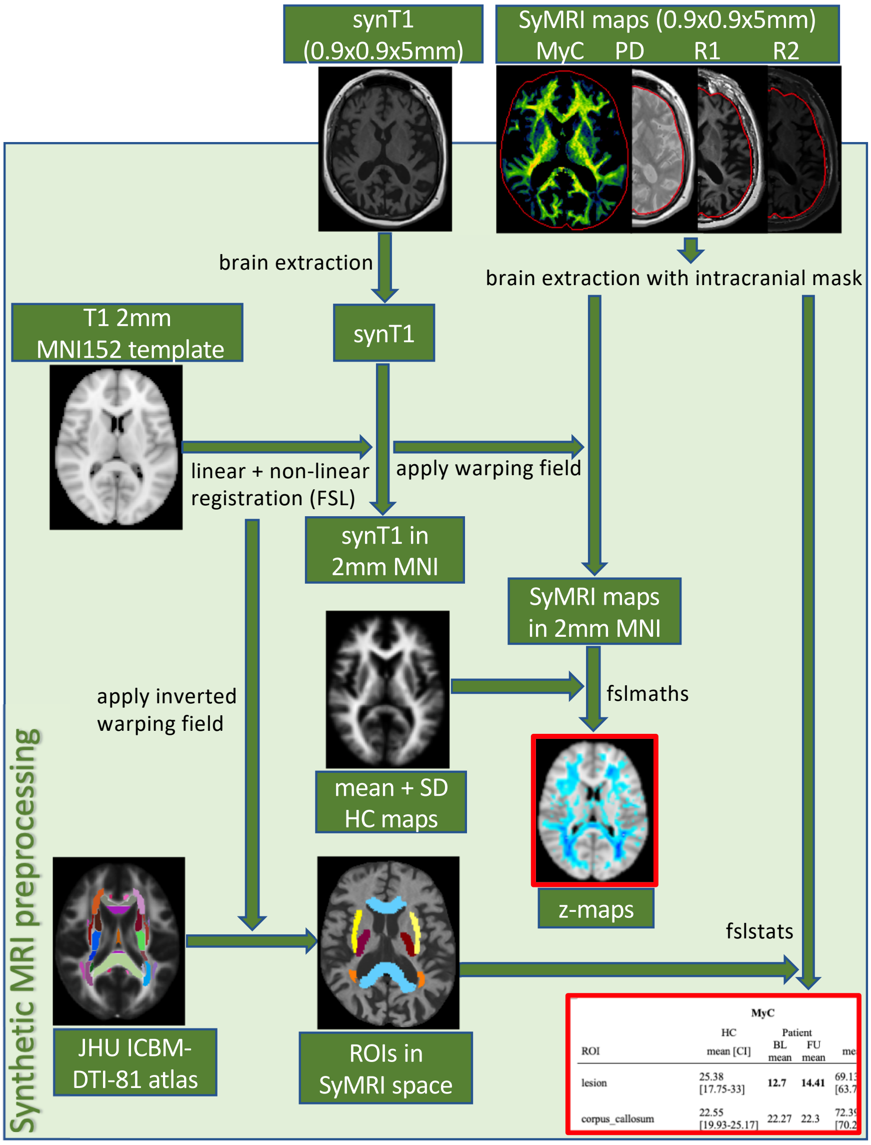

Detailed description of MRI preprocessing

DTI preprocessing

MRI data was converted into Nifti format using *dcm2niix* (Chris Rorden's dcm2niiX version v1.0.20200331) [1]. High resolution 3D T1 weighted images from 3 T MRI were preprocessed by alignment to anterior and posterior commissure, cropping (FSL's robustfov [2]), intensity normalization and brain extraction (using Freesurfer's *autorecon1* pipeline [3]) to optimize subsequent registration. After preprocessing of the diffusion images including denoising, Gibb's ringing correction, and bias field correction, which was conducted by use of the software MRtrix [4], non-linear registration of the preprocessed b0 image to the preprocessed T1 weighted

image was performed to account for susceptibility induced distortions [5]. For that, the T1 weighted image was resampled to a voxel size of 2 mm to reduce a possible bias by deformation to a resolution of 1 mm. Registration was conducted with ANTS registration pipeline *SyNQuick*, and the resulting transformation was applied to all diffusion images using *ANTSapplyTransforms* [6]. These preprocessing steps were performed on both the baseline and the follow-up images, whereby an additional rigid-body registration step (FMRIB's Linear Registration Tool - FLIRT) for the patient's follow-up data was done to align it to the baseline scan.

Subsequently, scalar diffusion tensor imaging (DTI) maps containing fractional anisotropy (FA), mean diffusivity (MD), radial diffusivity (RD; calculated by (λ_2 map + λ_3 map) /2), and axial diffusivity (AD; equals λ_1 map) were obtained by applying FSL's *DTIFIT*.

The resulting DTI maps were further edited using the first steps of the standard Tractography Based Spatial Statistics (TBSS) [7] pipeline in FSL. This included an additional pre-processing script to remove brain-edge artifacts and to zero the end slices, as well as a non-linear registration step to align each individual DTI map to the FMRIB58_FA target in standard MNI152 space. Since the final resolution is 1x1x1 mm by default, the resulting maps were down sampled to a voxel size of 2 mm for conformance with the other patient data. For the healthy control group, all maps were averaged using *fslmaths* to obtain a custom group template.

To create z-maps in MNI space, it was necessary to add an additional registration step for the patients' DTI data, because the registration to the FMRIB58_FA target within TBSS was not sufficient to achieve a good alignment to HC template. Thus, patients' 3D T1 weighted images were transformed into 2 mm MNI space with linear and non-linear registration (FSL's FLIRT and FNIRT). The resulting warping field was applied to the DTI maps. Finally, z-maps in 2 mm MNI space were obtained by calculation of $\frac{patient\ map - mean\ HC\ map}{SD\ HC\ map}$ with SD = standard deviation.

Synthetic MRI preprocessing

SyMRI maps for both time points (Myelin, PD, R1, R2 maps) and global measures (WM, GM, CSF, NON, MyC expressed as % from ICV) were generated automatically using the Synthetic MR Software. To apply the lesion mask of the baseline scan to the follow-up images, the patients' follow up images were aligned to the baseline maps using FMRIB's linear and non-linear registration tools (FLIRT and FNIRT).

To create z-maps, patient's SyMRI images were transformed into MNI152 space. For that, synthetic T1 weighted images were brain extracted using FSL's BET and resampled to 2 mm using FLIRT. They were then aligned to a 2 mm MNI template by use of FLIRT and FNIRT. The resulting warping fields were applied to synthetic MVF, PD, R1, R2 maps.

SyMRI images of the healthy control group were aligned to a 2 mm MNI template to create a study specific template. For that, 3D T1w images, which were additionally obtained from the 1.5T scanner protocol for the healthy controls*, and synthetic T1 images were resampled to 2 mm first. Second, synthetic T1w images were aligned to 3D T1w images (FLIRT, 6 DOF). Third, the warping field from registration of 3D T1w images to the MNI template (FNIRT) was applied to the synthetic T1w images.

Finally, z-maps were again obtained by calculation of $\frac{patient\ map - mean\ HC\ map}{SD\ HC\ map}$ with SD = standard deviation.

ROI based analysis

For quantification of DTI metrics, we extracted means and standard deviations for FA, MD, RD, and AD values for the whole brain and within selected regions of interest (ROI) which were derived from the JHU-ICBM-labels atlas. We included posterior thalamic radiation, corticospinal tract, cerebral peduncle, posterior limb of internal capsule, superior corona radiata, and corpus callosum as pathologically affected regions. In order to align the ROIs to DTI maps, FLIRT and FNIRT was used to register the FMRIB58_FA template to the patient's baseline FA map and the resulting warping field was then applied to the JHU-ICBM-labels atlas. ROI mean values of the patient and the healthy control group were obtained using *fslstats*.

For the assessment of mean MVF, PD, R1, and R2 derived from Synthetic MR maps, the T1 weighted images were registered to the structural T1 template in MNI152 space (FLIRT and FNIRT), and the resulting transformation was inverted and applied to the ROI maps of the JHU-ICBM-labels. For the resulting ROIs in SyMRI space, mean values of patients and the healthy control group were obtained using *fslstats*.

In addition, we extracted values within the patient's lesion area affected by the symmetrical paraventricular leukoencephalopathy. For that, the lesion map was automatically generated by the Synthetic MR Software, manually edited using FSLeyes to improve its accordance with the lesion based on the FLAIR sequence. The resulting lesion map was aligned to DTI maps, which were previously registered to 2 mm 3D T1 images, by applying the transformation from linear

* 192 sagittal slices, repetition time: 2.2 s, echo time: 2.88 ms, inversion time: 900 ms, acquisition matrix: 256x246, voxel size: 1x1x1mm^3

registration of synthetic T1 image to 2 mm 3D T1 image. To compare the affected area to normal references, we registered the lesion mask to MNI space by applying the warping field from previous registration to the MNI target. Lesion values were again extracted using *fslstats*.

References

1. Li X, Morgan PS, Ashburner J, Smith J, Rorden C. The first step for neuroimaging data analysis: DICOM to NIfTI conversion. *Journal of Neuroscience Methods*. 2016;264:47-56. doi:10.1016/j.jneumeth.2016.03.001
2. Smith SM, Jenkinson M, Woolrich MW, et al. Advances in functional and structural MR image analysis and implementation as FSL. *Neuroimage*. 2004;23 Suppl 1:S208-19. doi:10.1016/j.neuroimage.2004.07.051
3. Fischl B. FreeSurfer. *Neuroimage*. 2012;62(2):774-781. doi:10.1016/j.neuroimage.2012.01.021
4. Tournier JD, Smith R, Raffelt D, et al. MRtrix3: A fast, flexible and open software framework for medical image processing and visualisation. *Neuroimage*. 2019;202:116137. doi:10.1016/j.neuroimage.2019.116137
5. Wang S, Peterson DJ, Gatenby JC, Li W, Grabowski TJ, Madhyastha TM. Evaluation of Field Map and Nonlinear Registration Methods for Correction of Susceptibility Artifacts in Diffusion MRI. *Frontiers in Neuroinformatics*. 2017;11. doi:10.3389/fninf.2017.00017
6. Tustison NJ, Cook PA, Klein A, et al. Large-scale evaluation of ANTs and FreeSurfer cortical thickness measurements. *Neuroimage*. 2014;99:166-179. doi:10.1016/j.neuroimage.2014.05.044
7. Smith SM, Jenkinson M, Johansen-Berg H, et al. Tract-based spatial statistics: voxelwise analysis of multi-subject diffusion data. *Neuroimage*. 2006;31(4):1487-1505. doi:10.1016/j.neuroimage.2006.02.024
